# Oncogenic and tumor-suppressive roles of Lipocalin 2 (LCN2) in tumor progression

**DOI:** 10.32604/or.2024.051672

**Published:** 2025-02-28

**Authors:** BAOXING HUANG, ZICHANG JIA, CHENCHEN FU, MOXIAN CHEN, ZEZHUO SU, YUNSHENG CHEN

**Affiliations:** 1 Clinical Laboratory, Shenzhen Children’s Hospital, Shenzhen, 518038, China; 2 State Key Laboratory of Tree Genetics and Breeding, Co-Innovation Center for Sustainable Forestry in Southern China, Key Laboratory of Tree Genetics and Biotechnology of Educational Department of China, Key Laboratory of State Forestry and Grassland Administration on Subtropical Forest Biodiversity Conservation, College of Life Sciences, Nanjing Forestry University, Nanjing, 210037, China; 3 Department of Orthopaedics and Traumatology, School of Clinical Medicine, Li Ka Shing Faculty of Medicine, The University of Hong Kong, Hong Kong SAR, China

**Keywords:** Tumor progression, Gene regulation, Oncogenesis, Lipocalin 2 (LCN2)

## Abstract

Lipocalin-2 (LCN2) is a member of the lipocalin superfamily with multiple functions and can participate in the transport of a variety of small lipophilic ligands *in vivo*. LCN2 is significantly expressed in various tumors and plays an important role in regulating tumor cell proliferation, invasion, and metastasis. The specific actions of LCN2 in tumors may vary depending on the particular type of cancer involved. In this review, we provide an extensive overview of the transcriptional and post-transcriptional regulation of LCN2 in health and disease. Furthermore, we summarize the impact of LCN2 dysregulation in a broad range of tumors. Lastly, we examine the mechanisms of action of LCN2 during tumorigenesis, progression, and metastasis. Understanding the complex relationships between LCN2 and tumor development, progression, and metastasis is vital for advancing our knowledge of cancer biology, developing biomarkers for diagnosis and clinical decision-making, and creating therapeutic strategies to improve the management of patients with cancer.

## Introduction

In recent centuries, cancer has been regarded as one of the major global public health problems, posing a serious threat to human life and health [1,2]. Lipocalin-2 (LCN2), which was originally isolated from neutrophils and termed neutrophil gelatinase-associated lipocalin (NGAL), belongs to the lipocalin superfamily [3]. The lipocalin family shares the same three-dimensional structure in which a single β-barrel consisting of eight antiparallel strands forms a central groove capable of binding ligands of different sizes, shapes, and chemotypes [4]. LCN2 is a secreted lipocalin that acts through NGALR to regulate intracellular responses [5]. LCN2 is involved in several biological processes, including kidney morphogenesis, tissue involution, acute phase response, iron transportation, immune responses, and bone metabolism [6–9]. In this review, we focus on the transcriptional regulation of LCN2, the role of LCN2 in the progression of tumors, and the molecular mechanisms involved in LCN2-mediated tumorigenesis and metastasis.

## Transcriptional and Posttranscriptional Regulation of LCN2

The murine orthologue of LCN2 24p3 was first characterized in primary mouse kidney cell cultures treated with SV-40 [10]. After that, LCN2 was found to accumulate in a wide range of cancers. The transcriptional induction of LCN2 is mainly controlled by three transcription factors: nuclear factor kappa-light-chain-enhancer of activated B cells (NF-κB) [11], activating transcription factor 4 (ATF4) [12], and signal transducer and activator of transcription 3 (STAT3) [13]. Suppression of LCN2 expression is regulated by two trans-acting factors: hypermethylated-in-cancer (HIC1) [14] and nuclear factor of activated T cells 3 (NFAT3) [15]. In addition, methylation was also found to suppress LCN2 transcription. Apart from transcriptional regulation, LCN2 is also regulated by gene expression at the posttranscriptional (RNA) level, the most important of which is RNA splicing [16].

### Transcriptional induction of LCN2 by transcription factors

**Figure 1:**
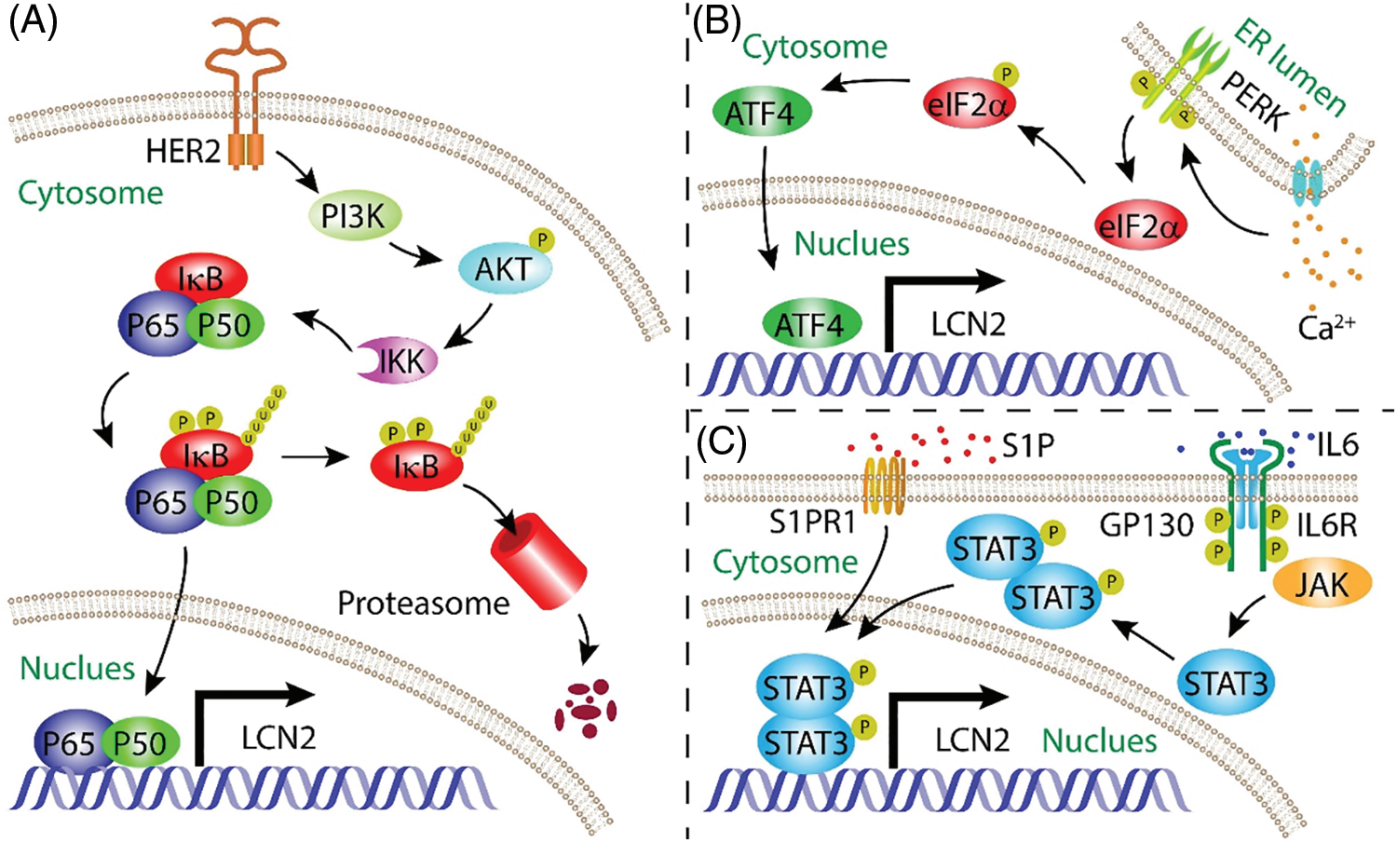
Mode of action summarizing the regulation of LCN2 expressi on by NF-κB, ATF4, and STAT3 transcription factors. (A) NF-κB signalling pathway induced LCN2 expression; (B) ER stress initiated ATF4 signalling pathway upregulated LCN2 expression in a Ca^2+^-dependent manner; (C) IL-6-stimulated STAT3-mediated signalling pathway increased LCN2 expression.

NF-κB is the first transcription factor reported to regulate LCN2 expression. Overexpression of epidermal growth factor receptor 2 (HER2) in breast cancer cells increases LCN2 expression [17]. Furthermore, the luciferase reporter assay showed that the accumulation of LCN2 contributed to transcriptional regulation of the promoter region with an NF-κB binding site consensus sequence [18]. Immunoprecipitation (ChIP) assays demonstrated the recruitment of the NF-κB subunits p65 and p50 to the LCN2 promoter [19]. Another study further showed that LCN2 accumulation is dependent on the activation of the HER2/PI3K/AKT/NF-κB cascade [20]. On the other hand, unfolded protein response (UPR)-induced ER stress is another inducer of LCN2 in an NF-κB-dependent manner [21] (Fig. 1A). Although the UPR is capable of activating the PI3K/AKT cascade, LCN2 expression is only partially modulated by this pathway [22], indicating that LCN2 is regulated by other transcription factors in the UPR.

ATF4 is a key transcription factor involved in the response to integrated stress, including ER stress [23]. Upon ER stress, ATF4 is another transcription factor that regulates LCN2 expression. The ISR pathway is a common adaptive pathway activated in response to a variety of stresses that regulates both survival and cell death in disease biology [24]. ER stress, viral infection, and nitrite deprivation can activate the endoplasmic reticulum stress kinase (PERK), pancreatic ER kinase (PKR), heme-regulated inhibitor (HRI), and general control nonderepressible 2 (GCN2) kinases. These kinases converge upon the phosphorylation of eIF2α, which leads to the global attenuation of Cap-dependent protein translation and the initiation of ISR-specific genes, including ATF4. ATF4 is the main effector of the ISR and controls the expression of genes involved in cellular adaptation [25]. In chronic kidney disease, the pathological cellular response to ER stress initiates ATF4 expression in a Ca^2+^-dependent manner (Fig. 1B). ATF4 functions as a transcription factor that induces LCN2 expression, leading to apoptosis. Inactivation of the LCN2 gene alleviates ER stress-induced apoptosis [26]. Uncontrollable growth of caners results in a wide range of stresses. ATF4 is a critical mediator of cancer survival or apoptosis in response to ISR [27]. However, the role of the ATF4/LCN2 axis in tumorigenesis, progression, and metastasis is still underaddressed.

STAT3 is the third transcription factor that directly induces LCN2 expression. This transcriptional regulation is stimulated by IL-6 [28]. In addition to autocrine signalling by tumor cells, paracrine signalling through stromal cells also plays an important role in the progression of tumors. For example, macrophages can be educated by secreted tumor factors to support the progression of tumors [29]. Sphingosine 1-phosphate (S1P) secreted by apoptotic breast cancer cells binds to S1PR1 on tumor-associated macrophages, which induces LCN2 expression in macrophage by activating the STAT3 pathway, promoting lymphangiogenesis and metastasis of breast cancer [30] (Fig. 1C).

### Transcriptional suppression of LCN2 by trans-acting factors

The accumulation of LCN2 in cancer not only contributes to the activation of transcriptional initiation factors but is also regulated by defects in its suppressors. The tumor suppressor gene HIC1 is deleted or silenced in a series of human cancers. When HIC1 expression is restored in breast cancer, elevated levels of LCN2 are antagonized by HIC1. HIC1 directly binds to the promoter of LCN2 to inhibit its transcription initiation, suppressing migration, invasion, and metastasis [14]. Moreover, LCN2 expression is negatively regulated by estrogen receptor alpha (ERα) in an NFAT3-dependent manner, inhibiting the migration of breast cancer cells [15].

### Transcriptional suppression of LCN2 by hypermethylation

DNA methylation is an important mechanism involved in transcriptional control and tumorigenesis [31]. Hypermethylation of the promoter region of LCN2 has been reported in breast cancer [32] and esophageal squamous cell carcinoma [33], but the underlying mechanisms are poorly understood.

### Posttranscriptional regulation of LCN2 by alternative splicing

As an important type of posttranscriptional regulation, splicing regulation is critical for gene expression. LCN2 contains seven transcript isoforms in the human genome, and genome-related studies have revealed a series of splicing mechanisms that contribute to tumorigenesis [34]. Forthermore, the LCN2 receptor NGLAR is also regualted by alternative splicing. NGALR-3 variants generated by alternative splicing in esophageal cancer may act as potential LCN2 receptors and play a role in LCN2-mediated iron transport in esophageal cancer [35].

## Roles of LCN2 in Tumorigenesis, Progression, and Metastasis

LCN2 has been suggested to regulate the development of a wide range of tumors [36,37]. However, the roles of LCN2 in tumorigenesis, progression, and metastasis vary from tumor to tumor. Here, we review the tumor-promoting and tumor-suppressing roles of LCN2.

### LCN2 promotes tumorigenesis, progression, and metastasis

LCN2 acts as an oncogene that promotes the progression of breast cancer [13] as well as, oral squamous cell carcinoma [38], myeloproliferative neoplasm [39], skin squamous cell carcinoma [40], gastric carcinoma [41], esophageal squamous cell carcinoma [42], thyroid cancer [43], and prostate cancer [44]. LCN2 was first reported to be upregulated in breast cancer tissues at both the mRNA and protein levels compared with normal tissues [45]. A later study of 207 breast cancer samples revealed that the LCN2 level is correlated with histological grade and metastasis, making it a potential prognostic marker [46]. To investigate the roles of LCN2 in breast cancer genesis and metastasis, genetic mouse models and mouse xenograft models have been widely employed. Inhibiting LCN2 in an MMTV-ERBB2 (V664E) mouse model significantly delayed mammary tumorigenesis and metastasis [20]. Suppression of primary mammary tumor formation has also been reported in MMTV-PyMT^129^ and MMTV-PyMT^B6^ mouse models with different LCN2 defects [47]. Apart from endogenous and autocrine LCN2, stromal-derived LCN2 significantly induces the dissemination of breast tumor cells into the lung in orthotopic mammary tumor mouse models [48]. The tumor-promoting role of LCN2 has also been reported in cancers other than breast cancer, e.g., stromal cell-derived LCN2 is associated with poor differentiation and prognosis in oral squamous cell carcinoma [49]. In thyroid cancer, the tumour-promoting effect of LCN2 was demonstrated in a nude mouse xenograft model [50]. In addition, LCN2-mediated tumourigenesis has been reported in human cutaneous squamous cell carcinoma xenograft models [40] and myeloproliferative tumour cell lines [18]. Moreover, *in vitro* studies have shown that LCN2 plays a positive role in the progression of prostate cancer [51]. In addition, expression level correlation analysis using immunohistochemistry, western blot and gelatin zymography have demonstrated that LCN2 is positively associated with invasion and poor progression in oesophageal squamous cell carcinoma and gastric cancer specimens [42,52].

### LCN2 suppresses tumorigenesis, progression, and metastasis

LCN2 is also known as a tumor suppressor gene that plays a repressive role in the progression of pancreatic cancer, ovarian carcinoma, and hepatocellular carcinoma. In ovarian carcinoma, immunoreactive LCN2 is associated with tumor grade and metastasis. LCN2 is not expressed in normal tissues, and a low level of LCN2 is detected in benign tissues. The highest LCN2 level was found in borderline and grade 1 ovarian carcinoma, while the LCN2 level decreased in advanced ovarian carcinoma. Therefore, LCN2 is a potential marker for monitoring the progression of ovarian cancer [53]. LCN2 is also reported to be an early diagnostic marker for pancreatic cancer. Similarly, LCN2 is highly expressed in early dysplastic lesions, while it is only expressed at low levels in healthy pancreatic tissue. In pancreatic cancer, LCN2 expression is correlated with tumor differentiation; well-differentiated and moderately differentiated tissues exhibit strong LCN2 expression, while poorly differentiated tissues are uniformly negative for LCN2 [54]. Moreover, elevated LCN2 expression suppressed the progression of pancreatic cancer in an orthotopic mouse model [55]. In hepatocellular carcinoma, LCN2 levels are positively associated with differentiation, and metastasis is suppressed by LCN2 [36].

### Paradoxical roles of LCN2 in tumorigenesis, progression, and metastasis

The role of LCN2 in the progression of tumors is dependent mainly on the type of tumor. However, paradoxical roles of LCN2 have been reported in colorectal cancer. The LCN2 level was positively associated with colorectal cancer stage and recurrence of stage II tumors, and LCN2 expression is an independent prognostic factor for overall survival [56]. Moreover, LCN2 is not a useful marker for the early diagnosis of malignant transformation [57]. In addition, a xenograft model showed that LCN2 promotes the progression of colorectal cancer [56]. Moreover, LCN2 exerted oncogenic effects on APCmin tumors, as evidenced by increasing intestinal tumor size [58]. In contrast, studies have shown that LCN2 plays a tumor-suppressive role in colorectal cancer. LCN2 defects increase tumor multiplicity, indicating that LCN2 is a tumor suppressor [58]. The tumor-suppressing role of LCN2 was further supported by an independent study that showed that the invasion and migration of colorectal cancer cells were suppressed by LCN2 [59].

### Mechanisms underlying LCN2-mediated tumorigenesis, progression, and metastasis

LCN2 is upregulated in a variety of tumors, and its expression is correlated with clinical outcomes. The correlation varied from tumor to tumor. We review the molecular mechanisms underlying the oncogenic and tumor-suppressive roles of LCN2.

### Mechanisms underlying LCN2-induced tumorigenesis, progression, and metastasis

Oncogenic roles of LCN2 is mediated by matrix metalloproteinase-9 (MMP9), stabization, induction of epithelial to mesenchymal transition (EMT), and DNA damage.

LCN2 exerts significant effects on the progression of tumors through stabilization of MMP9 [60]. MMP9 is a member of the matrix metalloproteinase family of extracellular proteases that plays an important role in angiogenesis and metastasis [61]. The LCN2/MMP9 complex was first isolated based on covalent binding in human neutrophils [62,63]. In addition, LCN2 has been reported to prevent the autodegradation of MMP9 *in vitro*, thus increasing its enzymatic activity upon covalent binding [64]. In a murine xenograft model, overexpression of LCN2 in the MCF-7 human breast cancer cell line increased MMP9 levels, which was accompanied by increased proliferation and angiogenesis [65]. In addition, the LCN2/MMP9 complex is present in the urine of 86.36% of patients with breast cancer but is not detected in healthy donors [65]. Moreover, the LCN2/MMP9 complex is correlated with worse overall survival in patients with gastric cancer [66].

LCN2 is a mediator of EMT that promotes breast cancer metastasis. EMT occurs during embryonic development, allowing cells to adapt to migratory and invasive behaviors. This process also occurs in tumor cells that undergo prometastatic transitions [67]. Overexpression of LCN2 in breast cancer, accompanied by increased expression of the key EMT transcription factor Slug, upregulated the mesenchymal markers vimentin and fibronectin while downregulated the epithelial marker E-cadherin, increasing the invasiveness of the tumor. The EMT reprogramming of breast cancer promoted metastasis in an orthotopic murine model [68]. Slug is also upregulated by LCN2 in an ERK signalling-dependent manner, promoting prostate cancer cell invasion and migration [69].

**Figure 2:**
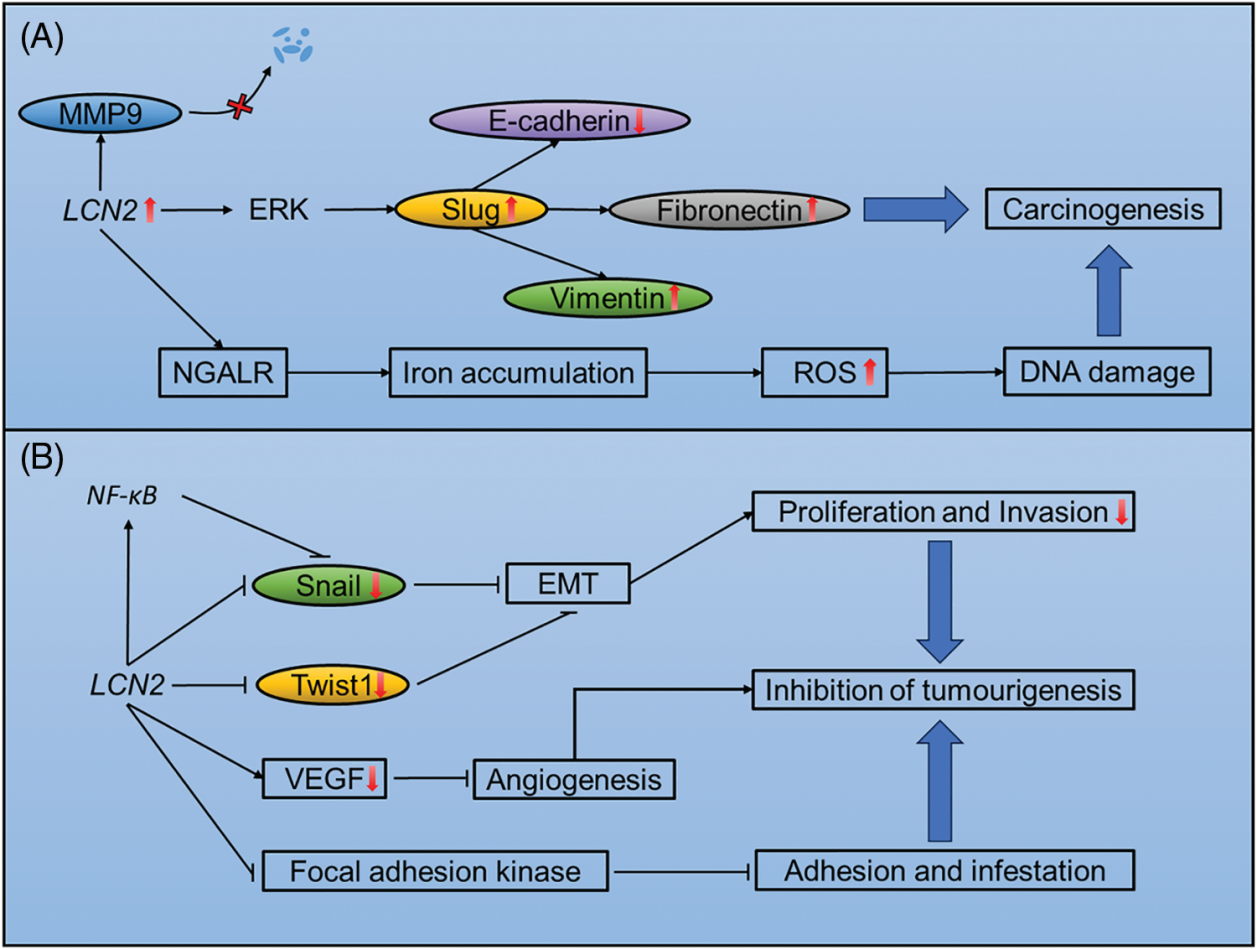
Mechanisms of LCN2-mediated tumourigenesis, progression and metastasis. (A) Potential mechanisms of LCN2-induced tumourigenesis, progression and metastasis. (B) Mechanisms of LCN2-mediated inhibition of tumourigenesis, progression and metastasis.

LCN2 is involved in DNA damage that contributes to the progression of tumors. One of the most important functions of LCN2 is regulating iron metabolism. When loaded with iron, LCN2 is endocytosed through NGALR, increasing intracellular iron levels [4]. Paracrine secretion of LCN2 by JAK2V617F^+^ cells increases intercellular iron levels in neighboring normal cells, resulting in elevated ROS levels. Excessive ROS levels, in turn, cause DNA damage and genetic instability, increasing the risk for leukemic transformation [70], although increasing intracellular iron accumulation arrests cell growth and even results in apoptosis [70,71]. Moreover, iron uptake via LCN2 reportedly regulates the survival of thyroid tumor cells [72]. The iron scavenging effect of LCN2 is involved in increased tumor size in the distal small intestine [58]. Detailed mechanisms of LCN2-induced tumourigenesis, progression and metastasis are shown in Fig. 2A.

### Mechanisms underlying LCN2-mediated suppression of tumorigenesis, progression, and metastasis

Manipulation of EMT and iron transportation by LCN2 can also suppressive tumor progression. In addition, LCN2 significantly suppresses tumor progression through inhibition of EMT and angiogenesis.

LCN2 plays dual roles in the manipulation of EMT. On the one hand, LCN2 promotes EMT and distant metastasis. On the other hand, LCN2 is also capable of suppressing EMT to reduce tumor metastasis. The EMT-suppressive role of LCN2 has been found in hepatocellular carcinoma. When upregulated in SH-J1 cells, LCN2 reduces the expression of Twist1, a key transcription factor that regulates EMT and inhibits cell proliferation and invasion. Furthermore, the EMT inducers EGF and TGF-β barely changed the expression of EMT markers in LCN2-overexpressing SH-J1 cells [73]. In addition, LCN2 is suppressed by EGF in ovarian cancer cell lines undergoing EMT [74]. In colorectal cancer, LCN2 manipulates EMT by suppressing snail, another key transcription factor involved in EMT. LCN2 can also acts as a trans-acting element that binds to the promoter region of NF-κB and suppresses the NF-κB/snail axis, inhibiting EMT and metastasis [59]. In addition, negative regulation of EMT has been reported in colorectal carcinoma [75].

LCN2-regulated iron transport may also significantly inhibits tumorigenesis. LCN2 is a bacteriostatic agent that kills bacteria through siderophore-mediated iron scavenging [76]. In an IL10^(−/−)^ mouse model, LCN2 protected the intestines by inducing inflammation and inhibited tumorigenesis through alterating in the gut microbiota [77].

LCN2 is a regulator of angiogenesis during tumor progression. In pancreatic cancer, LCN2 overexpression downregulates VEGF production and inhibits angiogenesis. In addition, upregulation of LCN2 inhibits the phosphorylation of focal adhesion kinase, impairing tumor cell adhesion and invasion [55]. Detailed mechanisms of LCN2 inhibition of tumourigenesis, progression and metastasis are shown in Fig. 2B.

## Discussion

LCN2 is dysregulated in a variety of cell types, including tumor cells. The transcription of LCN2 is controlled by three key transcription factors, namely NF-κB, ATF4, and STAT3, a suppressor HIC, and hypermethylation. NF-κB is a well-known transcription factor that upregulates LCN2 levels in tumor cells under inflammatory conditions and ER stress [78], but the regulation of LCN2 by ATF4 and STAT3 in tumor biology has not yet been reported. IL-1β expression has been reported to be closely related to LCN2 levels in prostate cancer cells [51]. Moreover, IL-1β is also capable of activating the STAT3 transcription factor [79], suggesting that the induction of LCN2 through IL-1β is under the control of STAT3. However, the hypermethylation and transcriptional suppression of LCN2 are still poorly understood.

Oncogenic and tumor-suppressive roles of LCN2 have been reported in different types of tumors, regardless of the contradictory roles reported in colorectal cancer. During embryonic development, critical genes controlling developmental processes fluctuate along different developmental stages. With tumor progression resembling embryonic development, LCN2 serves as a key gene that regulates this process and plays different roles in different kinds of tumors and different progression stages. In this regard, the pattern of temporal LCN2 expression during tumor development indicates the role of LCN2. In terms of the tumor-suppressive role of LCN2 in pancreatic cancer, ovarian carcinoma, and hepatocellular carcinoma, LCN2 levels are extremely high during tumorigenesis and then decrease during tumor progression. In addition, LCN2 is associated with differentiation. Consistent with its expression pattern, LCN2 plays a tumor-suppressive role in the progression of tumors. On the other hand, the LCN2 level does not decrease during the progression of breast cancer, myeloproliferative neoplasms, skin squamous cell carcinoma, gastric carcinoma, esophageal squamous cell carcinoma, or thyroid cancer. In these cancers, LCN2 has oncogenic effects, promoting tumor progression. Interestingly, paradoxical roles have been reported in colorectal carcinoma. Guts are abundant with microbes that are directly involved in colorectal carcinogenesis [80]. In addition, LCN2 is a bacteriostatic agent that can moderate the microbiota and suppress carcinogenesis in the colon and rectum [77]. Therefore, the role of LCN2 in the progression of colorectal carcinoma could depend on bacterial strains present in the gut [77,81].

The roles of LCN2 in tumor biology are not fully understood. First, LCN2 might play a role in the dedifferentiation of tumors. LCN2 plays a tumor-suppressive role by inducing EMT in ovarian carcinoma and hepatocellular carcinoma, where LCN2 levels are associated with well differentiation [73,74]. In addition, EMT is a prometastatic process associated with dedifferentiation [67]. Therefore, LCN2 can inhibit dedifferentiation during tumor progression. In addition, the roles of LCN2 in different progression stages remain to be addressed. LCN2 is highly expressed in the early progression stage of pancreatic carcinoma [82] and ovarian carcinoma [74], making it a potential marker for early diagnosis and an indicator of malignant transformation. Although LCN2 reportedly inhibits the late-stage progression of angiogenesis and EMT in pancreatic carcinoma and ovarian carcinoma, respectively, a high LCN2 level in the early stage implies a role for LCN2 in tumorigenesis. Finally, the role of LCN2 as a trans-acting factor in transcriptional regulation is less understood. LCN2 is localized in both the nucleus and cytoplasm of Hep3B and THLE2 cells [73], indicating that LCN2 functions as a trans-acting element. Twist1 is a direct target of LCN2. In hepatocellular carcinoma, LCN2 inhibits the transcriptional initiation of Twist1 to moderate EMT and suppress metastasis [73]. In addition, the promoter activity of NF-κB is antagonized by LCN2, which suppresses the metastasis of colorectal cancer through the snail signalling pathway [59]. Chromatin immunoprecipitation (ChIP) is a powerful method for detecting target genes of trans-acting elements. Additional roles of LCN2 in transcriptional regulation could be revealed by profiling downstream target genes using ChIP.

## Conclusions

Taken together, the molecular mechanisms involved in the oncogenic and tumor-suppressive roles of LCN2 are associated with EMT, iron metabolism, the LCN2/MMP9 complex, and angiogenesis. EMT is a prometastatic signature indicating poor prognosis in patients with tumors. This transition is tightly regulated by LCN2. Induction of EMT promotes tumor progression, while suppression of EMT restricts tumor cells from distal dissemination. Similarly, iron metabolism also participates in both oncogenic and tumor-suppressive processes. On the one hand, an increase in the intracellular iron level caused by LCN2-mediated endocytosis elevates the ROS level, which, in turn, increases genetic instability and promotes tumor progression. On the other hand, iron metabolism regulated by LCN2 also plays a bacteriostatic role in innate immunity. Alteration of the microbiota by LCN2 protects the gut from tumorigenesis. Moreover, the stabilization of MMP9 plays an oncogenic role and suppresses angiogenesis, inhibiting metastasis.

## Data Availability

None.

## Abbreviations

ATF4: Activating transcription factor 4
ChIP: Chromatin immunoprecipitation
EMT: Epithelial to mesenchymal transition
Erα: Estrogen receptor alpha
HER2: Epidermal growth factor receptor 2
HIC1: Hypermethylated-in-cancer
LCN2: Lipocalin-2
MMP9: Metalloproteinase-9
NGAL: Neutrophil gelatinase-associated lipocalin
NFAT3: Nuclear factor of activated T cells 3
NF-κb: Nuclear factor kappa-light-chain-enhancer of activated B cells
ISR: Integrated stress response pathway
PERK: Endoplasmic reticulum stress kinase
PKR: Pancreatic ER kinase
HRI: Heme-regulated inhibitor
GCN2: General control nonderepressible 2
S1P: Sphingosine 1-phosphate
STAT3: Signal transducer and activator of transcription 3
UPR: Unfolded protein response
